# Neonate with congenital pulmonary airway malformation concurrent with enteric duplication cyst: a case report of a rare anomaly

**DOI:** 10.1093/jscr/rjad502

**Published:** 2023-09-15

**Authors:** Amit Krishnan, Nicholas Schmoke, Christopher Nemeh, Yeu Sanz Wu, Vincent Duron

**Affiliations:** Westchester Medical Center, School of Medicine, New York Medical College, Valhalla, NY 10595, United States; Division of Pediatric Surgery, Department of Surgery, Columbia University Vagelos College of Physicians and Surgeons/New York-Presbyterian Morgan Stanley Children's Hospital, New York, NY 10032, United States; Division of Pediatric Surgery, Department of Surgery, Columbia University Vagelos College of Physicians and Surgeons/New York-Presbyterian Morgan Stanley Children's Hospital, New York, NY 10032, United States; Division of Pediatric Surgery, Department of Surgery, Columbia University Vagelos College of Physicians and Surgeons/New York-Presbyterian Morgan Stanley Children's Hospital, New York, NY 10032, United States; Division of Pediatric Surgery, Department of Surgery, Columbia University Vagelos College of Physicians and Surgeons/New York-Presbyterian Morgan Stanley Children's Hospital, New York, NY 10032, United States

**Keywords:** neonate, duplication cyst, congenital pulmonary airway malformation, laparoscopic, thoracoscopic, pediatric surgery

## Abstract

A congenital pulmonary airway malformation (CPAM) occurring concurrently with an enteric duplication cyst is a rare anomaly. Definitive management for both abnormalities is usually surgical resection. We present the uncommon case of a neonate with a CPAM and ileal duplication cyst, including pre-natal and post-natal workup. The patient was brought to the operating room for laparoscopic duplication cyst excision at 3 months of age. The patient returned to the operating room for a thoracoscopic right lower lobectomy at five months of age. This case presents a rare congenital anomaly with the concurrent presentation of a CPAM and enteric duplication cyst, with both being successfully excised minimally invasively.

## Introduction

Congenital pulmonary airway malformations (CPAM) and enteric duplication cysts are congenital abnormalities of two independent organ systems—the lung and the gut. CPAMs are a heterogenous group of benign cystic pulmonary lesions and are the most common congenital lung anomaly [1]. Type II (bronchiolar) CPAMs, described in this case report, have a good prognosis, low malignant potential, and are often associated with other congenital anomalies [2].

Enteric duplication cysts are congenital cystic or tubular structures attached to a portion of the alimentary tract with an incidence of 1 in 4500 births [3]. They most commonly occur in the midgut, particularly the ileocecal region, but may be located anywhere in the gastrointestinal tract [3]. While these congenital abnormalities have been well-described individually, their coexistence is rare, with no established management guidelines. We present an uncommon case of a neonate with a CPAM and ileal duplication cyst, including pre-natal and post-natal workup and operative intervention.

### Case report

A 26-year-old female presented for a routine ultrasound at 20 weeks gestation, which demonstrated a right lung lesion suspicious for a CPAM with a CPAM volume ratio of 0.25. Additionally, a cystic structure was identified in the abdomen. A subsequent fetal MRI at 24 weeks gestation demonstrated a hyperintense lesion in the right lower lobe with cystic components and no identifiable feeding vessel, concerning for a CPAM or hybrid lesion. The abdominal mass was also redemonstrated as a unilocular cystic structure with a differential including enteric duplication cyst, choledochal cyst, mesenteric cyst, or lymphatic malformation. Subsequent ultrasounds showed no additional anatomic abnormalities or evidence of fetal hydrops. Fetal whole genome sequencing was completed with no abnormalities.

**Figure 1:**
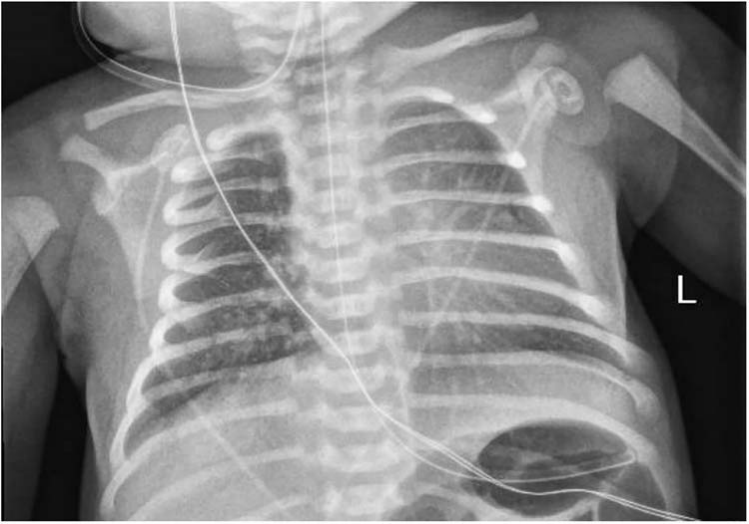
Chest x-ray with right medial lung opacity.

**Figure 2:**
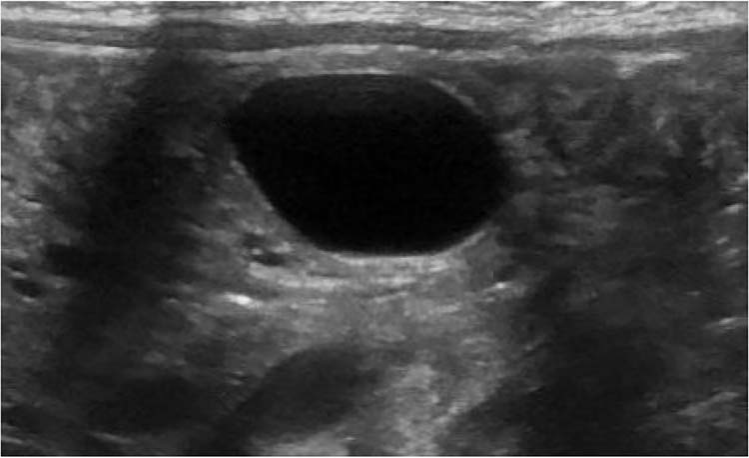
Ultrasound with enteric cyst with gut signature.

A 3.5 kg male was born via spontaneous vaginal delivery at 38 weeks. A chest radiograph demonstrated opacity at the medial right lung base (Fig. 1) consistent with the known congenital lung malformation. An abdominal ultrasound demonstrated a simple cyst with a gut signature consistent with an enteric duplication cyst (Fig. 2).

An additional abdominal ultrasound was obtained at two months of age, which redemonstrated a growing hypoechoic cystic structure with the persistence of a gut signature. A chest CT scan was obtained per institutional protocol for congenital lung malformations at ten weeks of age, which demonstrated an ill-defined area containing small cysts in the medial right lower lobe without systemic vascular supply or bronchial distribution consistent with a CPAM. Excision of both lesions was recommended, which the parents agreed to. The decision was made to proceed with laparoscopic duplication cyst resection followed by thoracoscopic right lower lobectomy 6 weeks later.

The patient underwent laparoscopic enteric cyst excision at 3 months of age. The cyst was found to be intimately connected to the mesentery of the ileum with no apparent connection to the bowel itself. It was carefully dissected with a laparoscopic LigaSure, without injury to the mesenteric vessels. The procedure was well tolerated, and the patient was discharged home on postoperative day 1. Pathology was consistent with an enteric duplication cyst.

The patient recovered well and was returned to the OR at 5 months of age for a thoracoscopic right lower lobectomy. The superior and basal segmental bronchi were identified and clipped. The basal bronchus and inferior pulmonary vein were stapled using vascular loads. The patient tolerated the procedure well and was discharged home on postoperative day 2. Surgical pathology was consistent with a CPAM, type 2.

## Discussion

This case presents a rare congenital anomaly with the concurrent presentation of a CPAM and enteric duplication cyst, with both being successfully excised minimally invasively. Individually, surgical excision for each anomaly has been established as optimal therapy to avoid subsequent complications, including the risk of infection, malignant transformation, or compensatory lung growth for CPAMs, and risk of abdominal pain, obstruction, bleeding, or cyst rupture for enteric duplication cysts [4, 5]. Due to the rarity of their concurrence, best practice in managing these anomalies is not well established.

While the pulmonary and enteric systems are independent organ systems, they originate from one common embryonic organ, the foregut. The respiratory system develops from an endodermal diverticulum arising from the ventral foregut, where the esophagus develops from the dorsal wall of the foregut [6]. This shared embryonic origin explains a concomitant pulmonary and esophageal anomaly. Dewberry et al. and Sun *et al.* [7, 8] reported cases of CPAMs and esophageal duplication cysts. Xu *et al.* [9] reported a case of CPAM concurrent with a gastric duplication cyst; both were managed with resection. This is the first report of a CPAM occurring concurrently with an ileal duplication cyst.

We decided to proceed with enteric duplication cyst excision first due to the more immediate risks associated with the duplication cyst. Once the patient had fully recovered and some time had elapsed between anesthetics, he was returned to the OR for CPAM excision. Both lesions were managed minimally invasively, with prior reports demonstrating the efficacy of minimally invasive surgery while avoiding the morbidity of an open procedure, including longer hospital stays, increased postoperative pain, a higher rate of wound infections, and worse cosmetic results [10–13]. Sukja *et al.* [11] presented a review of 35 children with enteric duplications cysts; 34 (97%) had their cysts approached minimally invasively, with only 3 (8%) requiring conversion to open operation. The timing of asymptomatic CPAM resection remains debated, with a recent Delphi process revealing that most expert surgeons believed that asymptomatic CPAMs should be resected at any point in the first year of life [14]. Additionally, a review of 237 children with asymptomatic CPAMs found a significantly lower rate of pulmonary infections in those who underwent resection at >2 years of age [15].

In summary, the results of the present study demonstrate a rare case of congenital CPAM presenting simultaneously with an ileal duplication cyst, with both being excised minimally invasively. This study highlights the workup and management of a rare anomaly that can help guide the treatment of patients presenting with similar pathology.

## Data Availability

This case report includes all relevant information; no additional data is available to share.
